# Clinical Characteristics of Virologically Confirmed Respiratory Syncytial Virus in English Primary Care: Protocol for an Observational Study of Acute Respiratory Infection

**DOI:** 10.2196/60669

**Published:** 2025-01-22

**Authors:** Uy Hoang, Utkarsh Agrawal, José Manuel Ordóñez-Mena, Zachary Marcum, Jennifer Radin, Andre Araujo, Catherine A Panozzo, Orsolya Balogh, Mihir Desai, Ahreej Eltayeb, Tianyi Lu, Catia Nicodemo, Xinchun Gu, Rosalind Goudie, Xuejuan Fan, Elizabeth Button, Jessica Smylie, Mark Joy, Gavin Jamie, William Elson, Rachel Byford, Joan Madia, Sneha Anand, Filipa Ferreira, Stavros Petrou, David Martin, Simon de Lusignan

**Affiliations:** 1 Clinical Informatics and Health Outcomes Research Group Nuffield Department of Primary Care Health Sciences University of Oxford Oxford United Kingdom; 2 Aetion, Inc New York, NY United States; 3 Moderna Cambridge, MA United States; 4 Moderna Biotech Distributor UK Ltd London United Kingdom; 5 Brunel University of London London United Kingdom; 6 Royal College of General Practitioners London United Kingdom

**Keywords:** infectious diseases, primary care, sentinel surveillance, point-of-care system, virologically, respiratory syncytial virus, acute respiratory infection, clinical characteristics, community dwelling, adult, vaccination, programme, united kingdom, incidence, elderly

## Abstract

**Background:**

There are gaps in our understanding of the clinical characteristics and disease burden of the respiratory syncytial virus (RSV) among community-dwelling adults. This is in part due to a lack of routine testing at the point of care. More data would enhance our assessment of the need for an RSV vaccination program for adults in the United Kingdom.

**Objective:**

This study aimed to implement point-of-care-testing (POCT) in primary care to describe the incidence, clinical presentation, risk factors, and economic burden of RSV among adults presenting with acute respiratory infection.

**Methods:**

We are recruiting up to 3600 patients from at least 21 practices across England to participate in the Royal College of General Practitioners Research Surveillance Centre. Practices are selected if they undertake reference virology sampling for the Royal College of General Practitioners Research Surveillance Centre and had previous experience with respiratory illness studies. Any adult, ≥40 years old, presenting with acute respiratory infection with onset ≤10 days, but without RSV within the past 28 days, will be eligible to participate. We will estimate the incidence proportion of RSV, describe the clinical features, and risk factors of patients with RSV infection, and measure the economic burden of RSV infection.

**Results:**

A total of 25 practices across different English health administrative regions expressed interest and were recruited to participate. We have created and tested an educational program to deploy POCT for RSV in primary care. In addition to using the POCT device, we provide suggestions about how to integrate POCT into primary care workflow and templates for high-quality data recording of diagnosis, symptoms, and signs. In the 2023-2024 winter RSV detection in the sentinel network grew between October and late November. According to data from the UK Health Security Agency, the peak RSV swab positivity was in International Standards Organization week 48, 2023. Data collection remains ongoing, and results from the subset of practices participating in this study are not yet available.

**Conclusions:**

This study will provide data on the RSV incidence in the community as well as rapid information to inform sentinel surveillance and vaccination programs. This information could potentially improve clinical decision-making.

**International Registered Report Identifier (IRRID):**

DERR1-10.2196/60669

## Introduction

### Background

Respiratory syncytial virus (RSV) infection in adults is reported to be a substantial burden to health care systems globally [1]. A recent systematic review of the disease burden of RSV in older adults in the United Kingdom reported an average of 175,070 primary care physician episodes and 7915 deaths per season attributed to RSV, equivalent to 1946 primary care physician episodes per 100,000 population and 88 deaths per 100,000 population older than 65 years of age [2,3]. Another meta-analysis reported that RSV caused 4.66% (95% CI 3.34-6.48) of symptomatic respiratory infections in annual studies and 7.80% (95% CI 5.77-10.45) in seasonal studies among adults older than 60 years [4].

However, RSV is not widely recognized as a cause of respiratory infections in adults as its clinical manifestations are often nonspecific [5-10]. Among those with predisposing factors, such as older age, weakened immune systems, or underlying cardiopulmonary conditions, RSV can increase the risk of developing serious acute lower respiratory tract infection (LRTI), cardiovascular sequela, and exacerbation of underlying conditions, all of which are associated with a significant risk of hospitalization and mortality [5,6,11].

Current guidelines do not recommend rapid diagnostic testing for respiratory viruses in primary care in England [12,13] and a recent scoping review showed the paucity of literature on studies of RSV tests in specific populations and settings [14] despite the difficulty of clinical diagnosis and the availability of a number of highly accurate point-of-care tests (POCT) platforms for RSV in the National Health Service (NHS) [9,15]. Recent studies have estimated that limited routine clinical testing can result in more than 100-fold underestimation of RSV incidence in laboratory surveillance studies [16]. RSV disease burden is of considerable interest because of the development of new vaccines and monoclonal therapies [17-19]. The UK’s Joint Committee on Vaccination and Immunization advised that an immunization program for RSV be established for older adults aged 75 years and older [17]. More precise contemporary data to estimate the incidence of RSV in the community and understand the clinical and economic burden of RSV infection in adults would assist in planning any new RSV vaccination program.

Deployed at scale, POCT for RSV has the potential to address the gaps in our knowledge about incidence, clinical presentation, and disease burden.

### Aim and Objectives

The aim of the Observational Study of Acute Respiratory Infection (ObservatARI) study is to deploy POCT in primary care clinics to provide data about the incidence, clinical presentation, risk factors, and economic burden of virologically confirmed RSV among older adults presenting with acute respiratory infection (ARI).

Our primary objective is to calculate the incidence proportion of virologically confirmed RSV among adults aged ≥40 years, in a primary care-based sentinel surveillance cohort overall and by age, sex, and subgroups of interest.

Secondary objectives include (1) examining the population-level incidence of RSV, (2) the incidence of RSV-LRTI, (3) use of secondary care, (4) clinical profile, and (5) comparison of clinical and economic burden in cases with and without RSV. Prespecified subgroups (sample size permitting) include age and sex.

## Methods

### Coordinating Center

The coordinating center for this study was the Nuffield Department of Primary Care Health Sciences, University of Oxford, Oxford, United Kingdom.

### Study Design

The ObservatARI study is ongoing, taking place between November 2023 and November 2024. It is nested within the English national sentinel network, the Oxford-Royal College of General Practitioners (RCGP) Research and Surveillance Centre (RSC), at the Nuffield Department of Primary Care, University of Oxford. This network of over 2000 primary care providers in England, referred to as general practices, is the primary care infectious disease surveillance network for England recruited to be representative of the English population [20]. It has been providing weekly data for over 57 years and is used to monitor trends in infectious disease and investigate real-world vaccine and treatment effectiveness [21].

All practices that contribute data to the RSC are eligible to participate in the ObservatARI study. We will prioritize those practices that currently undertake reference virology sampling for the sentinel network and have previous experience with undertaking respiratory illness studies, including POCT studies. In order to ensure geographic representation, up to 3 practices in each English health region (East of England, London, Midlands, North East, Yorkshire, North West, and South East and South West) have been selected to participate in the study [22]. These sites will receive training on the appropriate use of the POCT device from the manufacturer, which will be documented on a training log. Provision of the POCT device to these sentinel surveillance sites will allow for the collection of data on RSV, which is not routinely tested as part of usual care.

**Figure 1:**
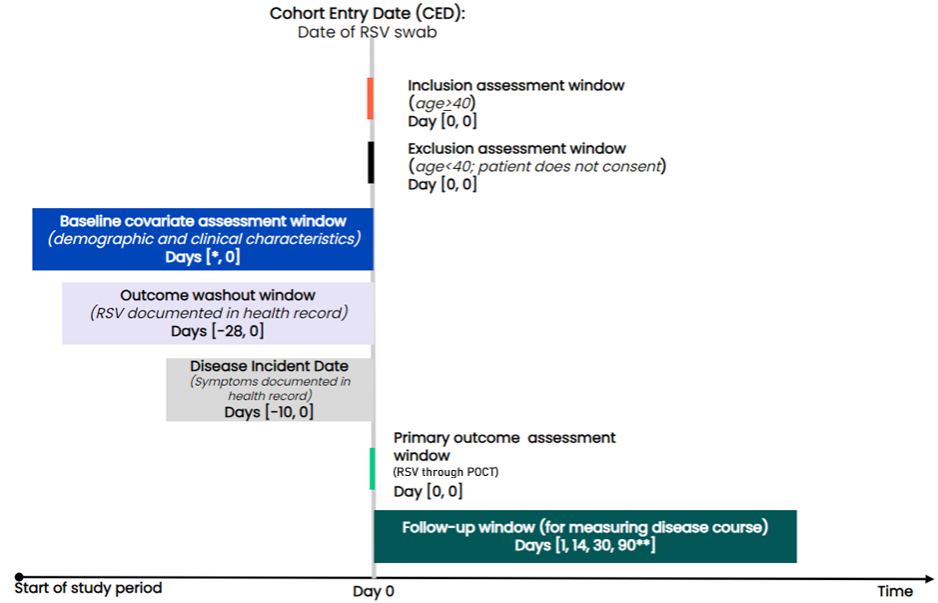
ObservatARI study design. ARI: acute respiratory infection; RSV: Research Surveillance Centre, POCT: point-of-care-testing. The study time period was from November 2023 to November 2024. *Lookback for baseline covariates will vary by variable using windows established by existing sentinel surveillance efforts. **Patients censored due to death or disenrollment from the clinic.

Figure 1 shows the cohort entry date of recruited patients is the date the POCT swab is taken and will serve as the study index date. Baseline covariates are assessed in the period of up to 5 years before cohort entry, and the precise lookback period will vary depending on the variable of interest.

We will follow-up enrolled patients for up to 14 days, 30 days, and 90 days for secondary outcomes of interest, such as clinical outcomes and health service usage. This information will be presented according to the test result from POCT (ie, RSV-positive and RSV-negative).

#### Case Definition of Eligible Patients

Fully registered primary care patients, of study practices, who present with ARI, with its onset in the last 10 days (including index date), where illness is not due to another plausible diagnosis, are eligible for enrollment in the ObservatARI study. These are the same criteria currently used by the UK Health Security Agency (UKHSA) sentinel surveillance system [23].

#### Sampling and Data Collection

We are undertaking opportunistic virology sampling [24], with potential participants being identified from fully registered patients and temporarily registered patients who present to the participating study practices with respiratory symptoms described in the case definition.

ObservatARI practices are advised to take 2 swabs, and 1 nasopharyngeal swab for testing using the Cepheid POCT analyzer. Nasopharyngeal swab samples have been studied and validated for the detection and quantification of respiratory viruses and are considered more accurate for RSV than nasal swabs [15,25,26]. The second one is a nasal swab for testing using the UKHSA National Virology Reference Laboratory as per Oxford-RCGP RSC’s standard practice [23].

Eligible patients are approached by their practice receive an explanation about the study and are asked for consent to take part when they present for a face-to-face consultation at the practice. Patients are informed that they are free to withdraw from the study at any time without giving any reason and without their legal rights being affected. Study participants will not receive compensation for taking part in this study.

After obtaining consent using a form with a prepopulated study number, the primary care physician or research nurse will take the virology swabs [25,26]. The nasopharyngeal swab is inoculated in a test kit and tested using the POCT analyzer as soon as possible after being taken. Only the patient study number is entered into the POCT machine.

We are using the Cepheid GeneXpert Xpress CoV-2, Flu, RSV plus POCT analyzer for this study. This multiplex polymerase chain reaction system tests for SARS CoV-2, influenza A, influenza B, and RSV. It has demonstrated excellent performance comparable to gold-standard laboratory assays with a sensitivity for RSV of 0.979 (95% CI 0.889-0.996) and specificity of 1.000 (95% CI 0.981-1.000) [27]. It has Conformité Européenne marking in the European Union. In the United States, it is approved by the US Food and Drug Administration and it is also waived for use by the Clinical Laboratory Improvement Amendments for rapid RSV testing. This study will not assess the accuracy of the POCT analyzer but will use the machine for its approved purpose.

The clinician is also asked to enter the following date of onset of four symptoms in the patient’s computerized medical record (CMR): (1) the presence or absence of fever because of infection, (2) cough (and if coughing is productive), (3) sore throat, and (4) shortness of breath or wheezing. In addition, the following four signs will be recorded in the CMR: (1) measured temperature (ear or tympanic preferred) ≥38 °C for fever, (2) peripheral oxygen saturation, (3) pulse rate >90 beats per minute for tachycardia, and (4) respiratory rate >20 breaths per minute for tachypnoea (Textbox 1). After the POCT analyzer has finished, the clinician is asked to code the result into the patient’s CMR.

**Figure 2:**
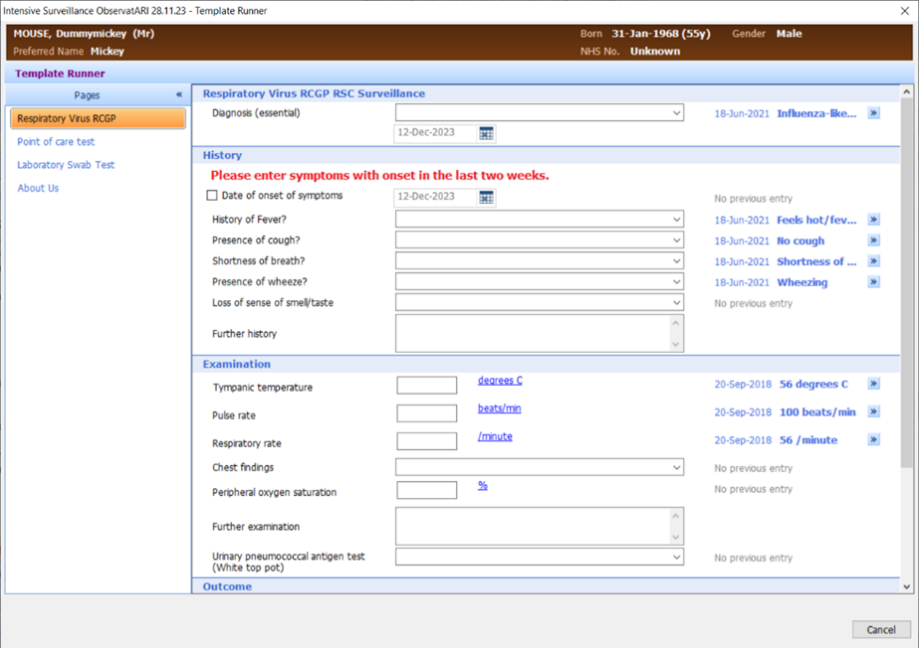
Sample ObservatARI data collection form. ARI: acute respiratory infection; RCGP: Royal College of General Practitioners; RSC: Research Surveillance Centre.

To facilitate the standardized collection of symptoms, signs, and POCT or laboratory results for the study, we have developed data entry templates for study practices using either the Egton Medical Information Systems or The Phoenix Partnership computerized medical systems available to participating practices (Figure 2). This allows clinicians in study practices to record respiratory symptoms and signs, and study consent, through dropdown menus and tick boxes. The results of POCT testing and reference laboratory testing will also be entered using these templates. The information from the templates is automatically entered into the patient’s CMR as a relevant Systemized Nomenclature of Medicine Clinical Terms, which will be extracted for data analysis as part of this study.

**Key data on symptoms and signs of acute respiratory infection.**

**Symptoms data**

- Presence or absence of fever because of infection
- Cough (and if coughing is productive)
- Sore throat
- Shortness of breath or wheezing

**Signs data**

- Measured temperature (ear or tympanic preferred) ≥38 °C for fever
- Peripheral oxygen saturation
- Pulse rate >90 beats per minute for tachycardia
- Respiratory rate >20 breaths per minute for tachypnea

### Data Sources

The Oxford-RCGP Clinical Informatics Digital Hub (ORCHID) trusted research environment will house the data required for this study.

We will use a pseudonymized NHS number to allow the linkage of ObservatARI data to national datasets. With appropriate approval, these data assets are held within the ORCHID trusted research environment. Data assets used for this study are as follows.

The primary virology diagnosis will be from POCT data collected from participating ObservatARI practices.

Supplemental data on test positivity will be available approximately a week later from the UKHSA reference laboratory data. In the event of a discrepancy between the POCT and UKHSA reference laboratory data, the POCT test will be selected as the main result with the proportion of discrepant results reported.

In addition, virology data from UKHSA virology sampling during the study period will be accessed to augment data obtained from POCT.

Pseudonymized patient data are also linked to UKHSA National Virology Reference Laboratory data results. NHS England national data collection provides access to hospitalization, intensive care unit admission, mortality, and other national datasets.

Hospital Episode Statistics (HES) is the transformed data, initially part of the Commissioning Data Set, covering patients attending accident and emergency units, admitted for treatment, or attending outpatient clinics at NHS hospitals in England, including details about length of stay and Health Care Resource Groups (HRGs) to enable health economic analysis. HRG consists of patient events that have been judged to consume a similar level of resource, which can be linked to an appropriate NHS tariff. Statistical controls have been applied to HES products. Data on cost will be extracted from HES using HRG. Health care resource use data during hospital admissions are not granularly available and, therefore, HRG codes will need to be used as a proxy for costing.

Emergency Care Data Set collects data from accident and emergency departments.

Civil Registrations include information including date, place, and certificated cause of death from the Office for National Statistics.

Data derived from primary and secondary care encounters are recorded using the Systemized Nomenclature of Medicine-Clinical Terms and *ICD-10* (*International Statistical Classification of Diseases, Tenth Revision*) codes, respectively [28]. The clinical concepts captured include diagnoses, therapy, test results, and other data [29]. Patient medical records in UK general practice have been computerized since the late 1990s, with pay-for-performance incentives introduced in 2004 for chronic disease management [30].

### Sample Size Calculation

Up to 3600 POCT swabs will be taken from consented, volunteer patients registered with the 21 ObservatARI general practices. Based upon the annual disease prevalence of 6% and reported sensitivity of 0.979 (95% CI 0.889-0.996) and specificity of 1.000 (95% CI 0.981-1.000) [27], we estimated the number of participants with and without RSV that would test positive and negative with the POCT if we sampled 3600 with ARI (Table 1).

**Table 1:** Expected counts (±95% CIs) for respiratory syncytial virus (RSV).

	RSV, expected count (95% CI)	No RSV, expected count (95% CI)	Total, n
Positive POCT^a^	211 (192-215)	0 (0-64)	211
Negative POCT	5 (1-24)	3384 (3320-3384)	3389
Total	216	3384	3600

^a^POCT: point-of-care-testing.

Given our estimates of prevalence, sensitivity, specificity, and the total available number of swabs in 21 practices in one season, we would expect to observe approximately 211 POCT true-positive RSV cases, which is sufficient to meet our primary objective of estimating the incidence proportion of POCT confirmed RSV.

We performed additional sensitivity analyses, keeping constant the number of swabs and prevalence of RSV, but assuming lower values of POCT sensitivity (90% and 80%) and estimated that 194 and 173 true-positive RSV cases would be detected, respectively. With smaller RSV case counts, SE of the incidence proportion (primary objective) would remain very similar and precise enough to generate meaningful inferences.

We have previously found that RCGP RSC practices using a POCT to diagnose respiratory viruses collect on average 6 swabs per practice per week over the winter season [31]. Thus, for our study in 21 practices over 12 months, we estimate that the target of 3600 swabs would be achievable.

### Covariate Ascertainment

Tables 2 and 3 show the covariates we will use for this study which are known to be associated with the incidence and severity of respiratory syncytial virus [32].

**Table 2:** Covariates associated with the incidence and severity of the respiratory syncytial virus.

Covariate	Categories
Age	As whole years 5-year increments (broader 10-year age bands may be considered)
Sex	Male or female
Ethnicity	White, Asian, Black, other, or mixed
Region	Divided into areas served by an integrated care system
Urban or rural designation	Urban or rural
Household composition	Divided into categories 1, 2, 3, 4-5, 6-10, 11, or more
Socioeconomic status will be measured using the index of multiple deprivation [32]	Quintiles from 1 (most deprived), 2, 3, 4, and 5 (least deprived)
BMI	Obese or not obese (BMI ≥ or <30), Also stratified as <18.5, 18.5-24.9, 25.0-29.9, 30.0-39.9, 40.0, or more)
Smoking status	Current smoker, previous smoker, or nonsmoker
Cambridge Multi-Morbidity Score	Quartiles No comorbidities, 1, 2, 3, 4, or more comorbidities
Comorbidities known to relate to increased risk of severe respiratory illness, or suboptimal vaccine response, or vaccine contraindication	Chronic respiratory disease Chronic obstructive pulmonary disease Chronic heart failure Chronic heart disease and vascular disease Chronic kidney disease Chronic liver disease Chronic neurological disease Diabetes mellitus Severe mental illness Morbid obesity Asplenia, or dysfunction of the spleen, immunosuppression due to disease, or treatment
Vaccination status within the season	Influenza vaccination COVID-19 vaccination Pneumococcal vaccination

**Table 3:** Covariates and categories.

Covariates	Categories
Age	As whole years 5-year increments (broader 10-year age bands may be considered)
Sex	Male or female
Ethnicity	White, Asian, Black, other, or mixed
Region	Divided into areas served by an integrated care system
Urban/rural designation	Urban or rural
Household composition	Divided into categories 1, 2, 3, 4-5, 6-10, 11, or more
Socioeconomic status will be measured using the index of multiple deprivation [32]	Quintiles from 1 (most deprived), 2, 3, 4, 5 (least deprived)
BMI	Obese or not obese (BMI ≥ or <30) Also stratified as <18.5, 18.5-24.9, 25-29.9, 30-39.9, 40, or more)
Smoking status	Current smoker, previous smoker, or nonsmoker
Cambridge Multi-Morbidity Score	Quartiles No comorbidities, 1, 2, 3, 4, or more comorbidities
Comorbidities known to relate to increased risk of severe respiratory illness or suboptimal vaccine response or vaccine contraindication	Chronic respiratory disease Chronic obstructive pulmonary disease Chronic heart failure Chronic heart disease and vascular disease Chronic kidney disease Chronic liver disease Chronic neurological disease Diabetes mellitus Severe mental illness Morbid obesity Asplenia or dysfunction of the spleen, immunosuppression due to disease, or treatment
Vaccination status within the season	Influenza vaccination COVID-19 vaccination Pneumococcal vaccination

### Statistical Analyses

For the primary objective, the incidence proportion of virologically confirmed RSV will be calculated as the number of swabs testing positive for RSV in the POCT, divided by the number of people fully registered at all 21 sites each month. Exact 95% CIs will be estimated using the Wilson method [33]. We will also calculate percent positivity as the number of positive cases divided by the number swabbed. Estimates will be presented overall and by key demographic and patient characteristics as defined above. Incidence proportions will be calculated by time (eg, moving average and monthly) depending on the sample size.

To approximate the response rate, and investigate potential selection bias, and external validity, we will compare the characteristics of patients for whom a swab was taken, with those who are ≥40 years presenting with an ARI code in the participating practices, and in the whole RSC network, respectively. This analysis will be restricted to the same study period.

For secondary objective 1, the incidence proportions (and 95% CI) estimated from the primary objective will be standardized (using the direct method) to the UK general population based on age or sex distribution [34].

For secondary objective 2, incidence proportions (and 95% CI) of RSV-LRTI will be calculated by dividing the number of cases meeting each of the three case definitions by the number of people at risk, for example, in a subgroup of interest.

For secondary objective 3, descriptive statistics (mean [SD], median [IQR], range [minimum and maximum], frequency [percentage]) will be used to describe the patient’s clinical features at presentation, for all patients and RSV-positive patients (eg, signs and symptoms; Textbox 1). For reference, the same information will be presented for patients with ARI who test negative for RSV. We will adjust for variables that are hypothesized to lead to the development of symptoms as well as RSV positivity. Crude and adjusted odds ratios and 95% CIs indicating the association between these variables and testing positive for RSV (vs testing negative) will be presented.

For secondary objective 4, descriptive statistics will be used to describe the patient covariates listed in Table 2. These covariate characteristics will be examined among all patients, with the information presented according to POCT status (ie, RSV-positive and RSV-negative). As in secondary objective 3, logistic regression will be used to estimate crude and adjusted odds ratios.

For secondary objective 5, the clinical and economic burden of RSV will be assessed by comparing the number of health care visits (eg, visits to the general practitioner, hospitalization, and intensive care unit visits), medication use (eg, antiviral and antimicrobial therapy), receipt of ventilation and supplemental oxygen use up to 6 months after the swab. Additional outcomes of interest will include primary care costs, prescription costs, medical test costs, and secondary care costs.

Our approach to the analysis will depend on the type of outcome variable. For instance, cumulative health care resource costs covering primary and secondary health care services for the cohorts will be calculated for each patient by attaching unit costs to each service encounter. This will enable us to present initial estimates of the costs associated with health care usage across each group in terms of means, SD, median, IQR, minimum, and maximum. We will examine the distribution of the health care costs to identify outliers. This information will be presented according to POCT status (ie, RSV-positive and RSV-negative) and further, by the subgroup of interest. These steps will ensure that we fully comprehend the nature of the cost data [35].

### Ethical Considerations

Data for the study are held on dedicated secure servers within the ORCHID TRE. The Research Group’s secure network is sited behind a firewall within the University’s network. Only staff members or associated members of the Research Group who have been appropriately trained and approved by the Head of Department can access the data from secure workstations or secure laptops with encrypted drives. All staff members of the Research Group working within the team base work from secure workstations or secure laptops with encrypted drives within the Research Group’s secure network. The staff of the study sponsor will be provided with aggregate-level study data only. A risk assessment of the physical security of the Research Group’s offices and server room has been conducted by the Building and Facilities Manager, the Faculty IT Service Manager, and the Research Group’s Information Governance Lead. The University is compliant with the Data Protection Act and UK General Data Protection Regulation and has systems for technical and organizational controls for information security, including a University-level Information Security and Governance Group, chaired by the University Senior Information Risk Owner. The Research Group’s private network has its own system-level security policy and is tested for vulnerabilities annually.

Study practices are given a stipend to cover the costs of training staff members and hosting the study. A small remuneration is also provided to practices for each POCT swab and reference laboratory virology sample taken to cover the additional time taken during each consultation to undertake swabbing for this study. Patients are not remunerated for taking part in this study.

The study received ethical approval for England and Wales from the Health Research Authority and Health and Care Research Wales on September 14, 2023 (Integrated Research Application System project ID: 329790, south central Oxford A Research Ethics Committee reference number 23/SC/0320).

The study and data extract were approved by the ORCHID Caldicott Guardian, the Primary Care Hosted Research Data sets Independent Scientific Committee of the Nuffield Department of Primary Care Sciences at the University of Oxford on November 7, 2023 (application reference PD-0030-2023).

### Protocol Amendments

Important protocol amendments will be referred to the English National Research Ethics Committees for ethical approval. Once approved, it will be communicated directly with the recruiting study practices. The amended protocol will be shared with all relevant parties, for example, investigators and clinical research networks in a timely manner.

## Results

We have received expressions of interest from 25 practices and have purposively recruited 21 practices from across the English health administrative regions.

**Figure 3:**
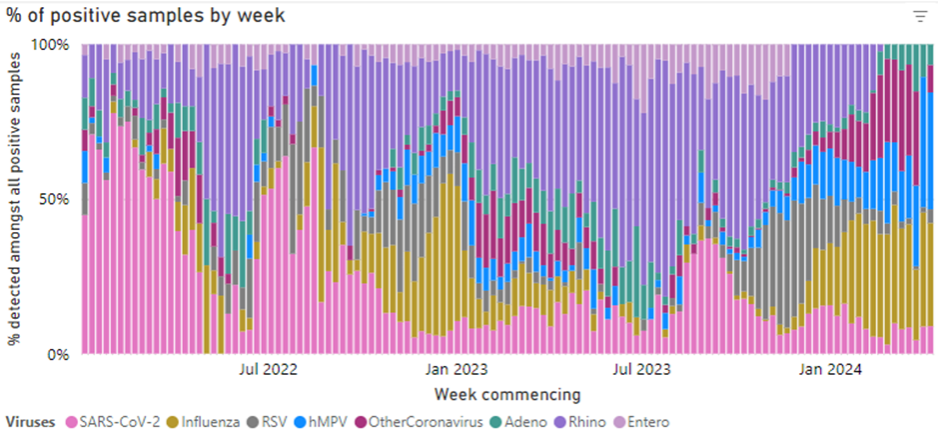
The UK Health Security Agency virology swabbing positivity in 2023-2024 [36].

In the 2023-2024 winter season, RSV detection in the sentinel network grew between October and late November. According to data from the UKHSA, the peak RSV swab positivity was in International Organization for Standardization week 48, 2023 (Figure 3 [36]). Data collection remains ongoing, and results from the subset of practices participating in this study are not yet available.

## Discussion

### Anticipated Findings

This study will generate contemporary data about the clinical presentation and disease burden of RSV among middle-aged and older adults in the community. Our primary outcome is the incidence of virologically confirmed, symptomatic, RSV infection presenting to primary care providers in England.

This study has a number of limitations. First, even with a sampling of 3600 eligible patients with ARI, it is expected that this study will only yield approximately 200 positive cases of RSV with the POCT. While this would be enough to estimate the incidence of RSV cases presenting to primary care with sufficient precision, the low number of cases could impact the number of covariates that we can adjust for in secondary objectives, as well as the estimation of ORs for categories with low frequencies (eg, underweight BMI category) and our ability to conduct subgroup analysis.

Second, there is inherent variation in clinical assessment and, thus, the decision to swab an eligible participant and exclude anyone with an illness due to another plausible diagnosis may potentially introduce bias. To minimize this variation, we will have robust site training available at the start of the study and throughout for new members of staff. We will also quantify any individual selection bias by comparing the characteristics of eligible swabbed and unsampled patients in POCT practices.

Third, a limitation of our opportunistic sampling strategy is that we will sample both fully registered and temporarily registered patients from study practices with eligible symptoms. Temporarily registered patients may include people at increased risk of infection or severe consequences of infection as well as people with less available information in their medical records such as refugee populations. We will quantify the effect this may have by comparing the characteristics of fully registered and temporarily registered patients in POCT practices.

Fourth, the study practices are not randomly selected so the results from the POCT surveillance cohort will not generalize beyond the selected clinics. To quantify any practice selection bias, we will compare the characteristics of patients for whom a swab was taken, with those presenting with an ARI code in the 21 participating practices, and in the whole RCGP RSC network, respectively.

Finally, the use of nasopharyngeal sampling is invasive and uncomfortable for the patient and it requires a trained provider to perform the collection. This may negatively impact the sensitivity of POCT in this older adult population, potentially biasing incidence estimates downwards. However, nasopharyngeal swabs have been validated for the detection and quantification of respiratory viruses [25,26]. They are considered the most accurate method for sampling RSV and practices in the Oxford-RCGP RSC sentinel network have many years of experience with undertaking nasopharyngeal swabs as part of national virological respiratory disease surveillance, which should reduce this risk [21,25,26]. In addition, where the virus has progressed lower in the respiratory tract, a nasopharyngeal swab may not detect these cases and a sputum sample may be required.

The limitations of the study must be considered in the context of its strengths. First, the study is nested within the English sentinel network, which includes participating practices that have developed expertise in identifying respiratory infections for the past 5 decades [21] and have a robust reporting infrastructure that supports rapid, accurate respiratory disease surveillance.

Second, the study uses a robust reverse transcription-polymerase chain reaction multiplex POCT platform that is rapid, accurate, cost-effective, simple to use, and reliable, and will provide viral positivity results for RSV, influenza, and SARS-CoV-2.

Third, the study leverages the NHS data system and a broader network of virology testing within the sentinel surveillance network to expand the breadth of the dataset (eg, clinical, economic, and humanistic variables) and ensure broader applicability of findings. Thus, we will have the opportunity to leverage the broader dataset of laboratory results and other data from patients from outside the 21 clinical sites in the analysis as needed.

### Conclusion

We have started to deploy POCT for RSV in primary care and believe that if done at scale, POCT may provide data on the RSV incidence in the community as well as rapid information to inform sentinel surveillance. This information may also provide data to inform decisions about the benefits of a UK-wide RSV vaccination program for the adult population.

## Abbreviations

ARI: acute respiratory infections
CMR: computerized medical record
HES: hospital episode statistics
HRG: health care resource groups
ICD-10: International Statistical Classification of Diseases, Tenth Revision
LRTI: lower respiratory tract infection
NHS: National Health Service
ORCHID: Oxford-RCGP Clinical Informatics Digital Hub
POCT: point-of-care test
RCGP: Oxford-Royal College of General Practitioners
RSC: Research and Surveillance Centre
RSV: respiratory syncytial virus
UKHSA: UK Health Security Agency

## Data Availability

The datasets generated during and/or analyzed during this study are available from the corresponding author on reasonable request and with permission of RCGP RSC [37].

## References

[1] Grace M, Colosia A, Wolowacz S, Panozzo C, Ghaswalla P (2023). Economic burden of respiratory syncytial virus infection in adults: a systematic literature review. J Med Econ.

[2] Wilkinson T, Beaver S, Macartney M, McArthur E, Yadav V, Lied-Lied A (2023). Burden of respiratory syncytial virus in adults in the United Kingdom: a systematic literature review and gap analysis. Influenza Other Respir Viruses.

[3] Sharp A, Minaji M, Panagiotopoulos N, Reeves R, Charlett A, Pebody R (2022). Estimating the burden of adult hospital admissions due to RSV and other respiratory pathogens in England. Influenza Other Respir Viruses.

[4] Nguyen-Van-Tam JS, O'Leary M, Martin ET, Heijnen E, Callendret B, Fleischhackl R, Comeaux C, Tran TMP, Weber K (2022). Burden of respiratory syncytial virus infection in older and high-risk adults: a systematic review and meta-analysis of the evidence from developed countries. Eur Respir Rev.

[5] Nam H, Ison MG (2021). Respiratory Syncytial Virus. Semin Respir Crit Care Med.

[6] Walsh EE, Peterson DR, Falsey AR (2004). Risk factors for severe respiratory syncytial virus infection in elderly persons. J Infect Dis.

[7] Andeweg SP, Schepp RM, van de Kassteele J, Mollema L, Berbers GAM, van Boven M (2021). Population-based serology reveals risk factors for RSV infection in children younger than 5 years. Sci Rep.

[8] Wang D, Cummins C, Bayliss S, Sandercock J, Burls A (2008). Immunoprophylaxis against respiratory syncytial virus (RSV) with palivizumab in children: a systematic review and economic evaluation. Health Technol Assess.

[9] Guidance (2021). Respiratory syncytial virus (RSV): symptoms, transmission, prevention, treatment.

[10] Oshansky CM, Zhang W, Moore E, Tripp RA (2009). The host response and molecular pathogenesis associated with respiratory syncytial virus infection. Future Microbiol.

[11] Woodruff RC, Melgar M, Pham H, Sperling LS, Loustalot F, Kirley PD, Austin E, Yousey-Hindes K, Openo KP, Ryan P, Brown C, Lynfield R, Davis SS, Barney G, Tesini B, Sutton M, Talbot HK, Zahid H, Kim L, Havers FP, Respiratory Syncytial Virus Hospitalization Surveillance Network (RSV-NET) (2024). Acute cardiac events in hospitalized older adults with respiratory syncytial virus infection. JAMA Intern Med.

[12] Barr R, Green CA, Sande CJ, Drysdale SB (2019). Respiratory syncytial virus: diagnosis, prevention and management. Ther Adv Infect Dis.

[13] (2023). NICE guideline [NG237]. Suspected acute respiratory infection in over 16s: assessment at first presentation and initial management.

[14] Bernstein D, Mejias A, Rath B, Woods CW, Deeter JP (2023). Summarizing study characteristics and diagnostic performance of commercially available tests for respiratory syncytial virus: a scoping literature review in the COVID-19 era. J Appl Lab Med.

[15] Onwuchekwa C, Moreo LM, Menon S, Machado B, Curcio D, Kalina W, Atwell JE, Gessner BD, Siapka M, Agarwal N, Rubbrecht M, Nair H, Rozenbaum M, Aponte-Torres Z, Vroling H, Begier E (2023). Underascertainment of respiratory syncytial virus infection in adults due to diagnostic testing limitations: a systematic literature review and meta-analysis. J Infect Dis.

[16] Rozenbaum MH, Judy J, Tran D, Yacisin K, Kurosky SK, Begier E (2023). Low levels of RSV testing among adults hospitalized for lower respiratory tract infection in the United States. Infect Dis Ther.

[17] (2023). Respiratory syncytial virus (RSV) immunisation programme for infants and older adults: JCVI full statement, 11 September 2023. 2023, Department of Health and Social Care.

[18] Walsh EE, Pérez Marc G, Zareba AM, Falsey AR, Jiang Q, Patton M, Polack FP, Llapur C, Doreski PA, Ilangovan K, Rämet M, Fukushima Y, Hussen N, Bont LJ, Cardona J, DeHaan E, Castillo Villa G, Ingilizova M, Eiras D, Mikati T, Shah RN, Schneider K, Cooper D, Koury K, Lino M, Anderson AS, Jansen KU, Swanson KA, Gurtman A, Gruber WC, Schmoele-Thoma B, RENOIR Clinical Trial Group (2023). Efficacy and safety of a bivalent RSV prefusion F vaccine in older adults. N Engl J Med.

[19] (2023). AstraZeneca to acquire Icosavax, including potential first-in-class RSV and hMPV combination vaccine with positive Phase II data.

[20] Leston M, Elson W H, Watson C, Lakhani A, Aspden C, Bankhead CR, Borrow R, Button E, Byford R, Elliot AJ, Fan X, Hoang U, Linley E, Macartney J, Nicholson BD, Okusi C, Ramsay M, Smith G, Smith S, Thomas M, Todkill D, Tsang RS, Victor W, Williams AJ, Williams J, Zambon M, Howsam G, Amirthalingam G, Lopez-Bernal J, Hobbs FDR, de Lusignan S (2022). Representativeness, vaccination uptake, and COVID-19 clinical outcomes 2020-2021 in the UK Oxford-royal college of general practitioners research and surveillance network: cohort profile summary. JMIR Public Health Surveill.

[21] de Lusignan S, Correa A, Smith GE, Yonova I, Pebody R, Ferreira F, Elliot AJ, Fleming D (2017). RCGP research and surveillance Centre: 50 years' surveillance of influenza, infections, and respiratory conditions. Br J Gen Pract.

[22] Regional teams.

[23] de Lusignan S, Hoschler K (2023). UKHSA Surveillance Commissioning Letter 2023.

[24] Andrade C (2021). The inconvenient truth about convenience and purposive samples. Indian J Psychol Med.

[25] Debyle C, Bulkow L, Miernyk K, Chikoyak L, Hummel KB, Hennessy T, Singleton R (2012). Comparison of nasopharyngeal flocked swabs and nasopharyngeal wash collection methods for respiratory virus detection in hospitalized children using real-time polymerase chain reaction. J Virol Methods.

[26] Munywoki PK, Hamid F, Mutunga M, Welch S, Cane P, Nokes DJ (2011). Improved detection of respiratory viruses in pediatric outpatients with acute respiratory illness by real-time PCR using nasopharyngeal flocked swabs. J Clin Microbiol.

[27] Cepheid xpert xpress CoV-2/Flu/RSV plus. Xpert xpress CoV-2/Flu/RSV plus - health care provider fact sheet.

[28] Systematized Nomenclature of Medicine Clinical Terms (SNOMED-CT).

[29] de Lusignan S (2005). Codes, classifications, terminologies and nomenclatures: definition, development and application in practice. Inform Prim Care.

[30] de Lusignan S, van Weel C (2006). The use of routinely collected computer data for research in primary care: opportunities and challenges. Fam Pract.

[31] de Lusignan S, Hoang U, Liyanage H, Tripathy M, Yonova I, Byford R, Ferreira F, Diez-Domingo J, Clark T (2020). Integrating molecular point-of-care testing for influenza into primary care: a mixed-methods feasibility study. Br J Gen Pract.

[32] (2019). English indices of deprivation 2019.

[33] Wilson EB (1927). Probable Inference, the Law of Succession, and Statistical Inference. Journal of the American Statistical Association.

[34] (2023). Population estimates for England and Wales: mid-2022.

[35] Sun T, Aroke H, Kogut S, Katenka N, Bratberg J, Buchanan A (2022). Early buprenorphine-naloxone initiation for opioid use disorder reduces opioid overdose, emergency room visits, and healthcare cost compared to late initiation. Am J Drug Alcohol Abuse.

[36] Virology Dashboard.

[37] Primary care hosted research data sets independent scientific committee (PrimDISC).

